# Chronic Otorrhœa

**Published:** 1872-03

**Authors:** David Webster

**Affiliations:** House Surgeon to Manhattan Eye and Ear Hospital, New York


### CHRONIC OTORRHŒA.

BY DAVID WEBSTER, M. D., HOUSE SURGEON TO MANHATTAN EYE AND EAR HOSPITAL, NEW YORK.

By otorrhœa is meant a discharge from the ear, and it is due to a suppurative inflammation, either of the external auditory canal, or the middle ear. In the vast majority of cases, the latter is the seat of the disease.

Chronic suppuration of the middle ear occurs much more frequently in children than in adults, and among its most prolific causes are the acute exanthemata.

The discharge is often very offensive, in smell, thus rendering the patient a nuisance, both to himself and to his friends. Polypi are often generated, and sometimes so increase in size as to protrude from the external meatus. The hearing is always more or less impaired, in some cases, going on to total deafness. The disease not very infrequently extends backward into the mastoid cells, causing severe pain, mastoid abscesses, and fistulous openings back of the ear.

In a certain proportion of cases the disease extends to the meninges and the brain. Several cases of death from this cause have been reported, and I have no doubt many other cases of the kind have *not* been reported, simply because the attending physician had not the slighest suspicion of the origin of the brain trouble. Notwithstanding the great inconvenience to which this disease subjects its victims, and the unpleasant and fatal consequences to which it is always liable to lead, physicians too frequently advise their suffering applicants for relief, to "let it alone." They tell the mother that to stop the discharge from her child's ear would injure his hearing, and would, perhaps, cause the disease to "strike in" and destroy the child's life.

It would be a waste of space for me to say anything in refutation of so absurd and preposterous an idea.

Chronic suppuration of the middle ear is often very difficult to cure, and yet I think I may safely venture to say that it can always be cured by appropriate and persevering treatment. I have seen a case under constant treatment, for over a year, finally cured. The patient had endured all the inconveniences of the discharge for over twenty years, hence the pertinacity with which it adhered to treatment.

The most important part of the treatment is careful and repeated cleansing. This is accomplished by syringing with warm water, and by the use of the cotton swab. A hard rubber syringe, with a blunt, rounded point, is the best, but an ordinary Davidson syringe answers the purpose very well. The auricle should be stretched upward and backward while syringing, in order to straighten the canal and allow the water to reach the middle ear.

After the ear is syringed until no more secretion comes out with the water, the suppurating surface should be carefully dried with the cotton swab, made by twisting a little Jeweller's cotton about the end of a dentist's cotton-holder, or, if this is not at hand, a slender bit of wood will do just as well.

The ear having been thoroughly cleansed in this manner, an astringent should be applied. I think a larger number of cases may be cured by a judicious use of nitrate of silver than by any other means.

Incline the head of the patient to one side, so that the middle ear may be at the most depending portion of the canal, drop into the meatus four or five drops of a solution of nitrate of silver, grains, forty—aquæ, ounces, one. Let it remain there a few seconds, so as to thoroughly apply itself to the whole of the diseased surface, then syringe it out with warm water. This should be done, at least three times a week, and always by the physician himself.

In many cases, a much stronger solution of nitrate of silver may be used. I have seen one application of a saturated solution of nitrate of silver cure a discharge from the middle ear, where weaker solutions had been used for months and failed. Where pain is caused by the application, it may always be quickly relieved by gently syringing with warm water.

In addition to this treatment by the physician, the patient's ear should be cleansed, by syringing, two or three times a day at home, and a solution of sulphate of zinc, from two to four grains to the ounce of water, should be poured into the ear, after cleansing, and allowed to remain about five minutes.

Meanwhile, if the physician has the proper apparatus and the necessary skill to use it, he should inflate the middle ear, by Politzer's method, or by the use of the eustachian catheter, three times a week, or oftener if convenient.

It would be an advantage to every physician to have ear-speculums and an ear-mirror, and to know how to use them. He would then be able to determine whether the discharge were from the middle ear, to ascertain the condition of the membrana tympani, and to watch the progress of the disease, and of its cure. He would, also, be able to tell whether the ear were perfectly cleansed.

